# Lignin Depletion Enhances the Digestibility of Cellulose in Cultured Xylem Cells

**DOI:** 10.1371/journal.pone.0068266

**Published:** 2013-07-18

**Authors:** Catherine I. Lacayo, Mona S. Hwang, Shi-You Ding, Michael P. Thelen

**Affiliations:** 1 Physical & Life Sciences Directorate, Lawrence Livermore National Laboratory, Livermore, California, United States of America; 2 Chemical and Biosciences Center, National Renewable Energy Laboratory, Golden, Colorado, United States of America; University of Massachusetts Amherst, United States of America

## Abstract

Plant lignocellulose constitutes an abundant and sustainable source of polysaccharides that can be converted into biofuels. However, the enzymatic digestion of native plant cell walls is inefficient, presenting a considerable barrier to cost-effective biofuel production. In addition to the insolubility of cellulose and hemicellulose, the tight association of lignin with these polysaccharides intensifies the problem of cell wall recalcitrance. To determine the extent to which lignin influences the enzymatic digestion of cellulose, specifically in secondary walls that contain the majority of cellulose and lignin in plants, we used a model system consisting of cultured xylem cells from *Zinnia* *elegans* . Rather than using purified cell wall substrates or plant tissue, we have applied this system to study cell wall degradation because it predominantly consists of homogeneous populations of single cells exhibiting large deposits of lignocellulose. We depleted lignin in these cells by treating with an oxidative chemical or by inhibiting lignin biosynthesis, and then examined the resulting cellulose digestibility and accessibility using a fluorescent cellulose-binding probe. Following cellulase digestion, we measured a significant decrease in relative cellulose content in lignin-depleted cells, whereas cells with intact lignin remained essentially unaltered. We also observed a significant increase in probe binding after lignin depletion, indicating that decreased lignin levels improve cellulose accessibility. These results indicate that lignin depletion considerably enhances the digestibility of cellulose in the cell wall by increasing the susceptibility of cellulose to enzymatic attack. Although other wall components are likely to contribute, our quantitative study exploits cultured *Zinnia* xylem cells to demonstrate the dominant influence of lignin on the enzymatic digestion of the cell wall. This system is simple enough for quantitative image analysis, but realistic enough to capture the natural complexity of lignocellulose in the plant cell wall. Consequently, these cells represent a suitable model for analyzing native lignocellulose degradation.

## Introduction

Deconstruction of the major plant cell wall polymers to small molecules is the essential first step in converting biomass to liquid biofuels. Biomass efficiently decomposes in nature through the synergistic activity of enzymes from microbial communities, which attack different components of the plant cell wall, ultimately resulting in carbon recycling [1]. The cell wall of higher plants is mainly composed of polysaccharides, including cellulose and hemicellulose, which are thought to intimately associate with lignin, a complex aromatic polymer characteristic of wall material known as secondary wall [2]. These durable organic polymers are collectively referred to as lignocellulose, and although their interactions within the cell wall are not well characterized, they play key roles in building an intrinsically resilient structure that is highly resistant to degradation [1,2].

Given the natural recalcitrance of the cell wall, much research focused on improving the efficiency of lignocellulose degradation towards the cost-effective production of biofuels [3,4]. Recalcitrance can be overcome through the removal or modification of wall components using a variety of pretreatments, which have been extensively utilized to achieve improved enzymatic digestion of plant biomass [5]. For instance, aqueous solutions of sulfuric acid [6] or acidified sodium chlorite [7] have been used to remove hemicellulose and lignin, respectively, as these polymers are barriers to cellulase activity [8,9]. Since plants synthesize lignin by polymerizing monolignol building blocks in a process that depends on reactive oxygen species (ROS) production, lignification can also be inhibited using chemical inhibitors of NADPH oxidase or ROS scavengers [10–12].

Studies examining the activity of cellulases have often relied on purified substrates that have dubious predictive value for the kinetic efficiency of enzymatic digestion of plant biomass during industrial biofuel production [13]. In contrast, plant tissue contains heterogeneous mixtures of cell types with varying wall composition [14], which can distort average or bulk analyses, complicate precise measurements at the single cell level, or undermine accurate statistical comparisons. We propose that an alternative to these substrates can be found in the secondary walls of xylem cells – also known as tracheary elements – which specifically differentiate in semi-synchrony from *Zinnia* *elegans* mesophyll cells during *in vitro* xylogenesis [15,16]. *Zinnia* xylem cells are easily recognized by microscopy [17,18] due to their prominent secondary wall thickenings, characteristic of xylem vasculature [15,19]. These secondary cell wall deposits primarily consist of parallel cellulose fibrils, hemicellulose, and also lignin, which is thought to provide mechanical strength [15,18,20]. Cultured *Zinnia* xylem cells are simpler than plant tissue, but more importantly, they possess the natural complexity of cell wall structures, making them suitable for *in vitro* studies of cell wall degradation. Using these cells, we focused on examining cellulose digestion, a process catalyzed by a variety of functionally distinct microbial endoglucanases and cellobiohydrolases, collectively known as cellulases [21].

To determine how cell wall deconstruction was influenced by lignin content, we examined the digestibility of cellulose in xylem cells following two different methods to deplete lignin: treatment with an oxidative chemical or with inhibitors of lignin biosynthesis. We then used microscopy to monitor cell wall degradation and a fluorescent cellulose-specific probe from *Clostridium thermocellum* (*Ct*CBM3-GFP) [22] to perform statistical comparisons of cellulose content and thus quantify the enzymatic removal of cellulose in populations of xylem cells. Since this probe is derived from a cellulose-degrading enzymatic complex and directly binds to cellulose, it was also used to assess cellulose accessibility and thus susceptibility to enzymatic attack. We show that lignin depletion in single xylem cells significantly increases cellulose digestibility by improving its accessibility, supporting observations of enhanced saccharification in transgenic plants with decreased lignification [23–25]. The current paucity of models providing the appropriate biological complexity of wall structure and quantitative methods for deconstruction has motivated this imaging-based approach, in which enzymatic efficiency is examined at the single cell level in a system that could be applied to biofuel research.

## Results

### Cultured xylem cells pretreated with acidified sodium chlorite are efficiently degraded by cellulase

**Figure 1:**
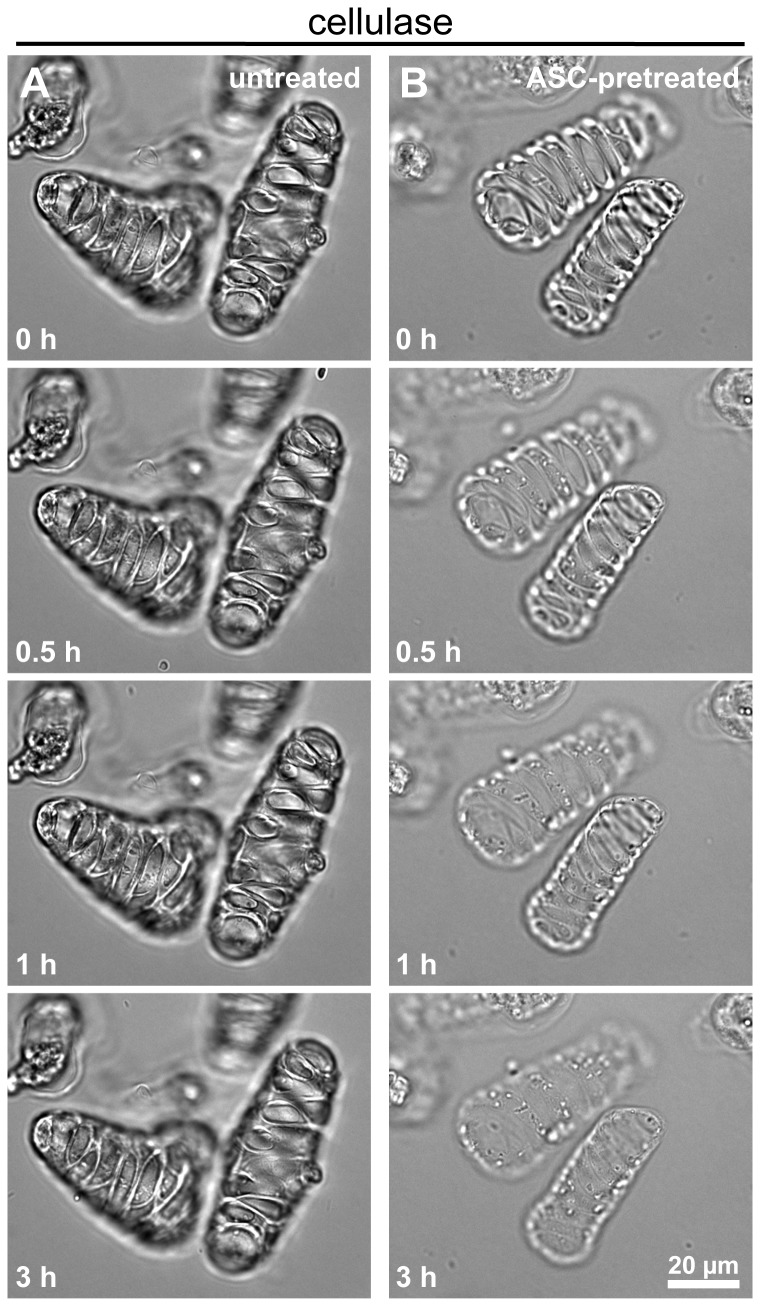
Pretreated xylem cells are efficiently degraded by cellulase. Time-lapse brightfield microscopy was used to image untreated (A) and ASC-pretreated xylem cells (B) incubated with cellulase. (A) Untreated xylem cells have no detectable changes in morphology during a 3-hour incubation. (B) Xylem cells pretreated with ASC are rapidly degraded with noticeable loss of material from the secondary cell wall thickenings within the first hour of incubation with cellulase. Scale bar=20 µm. Also see Video S1.

To test whether cultured plant cells would be amenable to enzymatic attack by exogenous enzymes, we isolated *Zinnia* xylem cells, which exhibit prominent wall thickenings (Fig. 1) composed mainly of secondary wall material. Using brightfield microscopy, we observed that untreated cells appeared intact for the entire duration of a 3-hour incubation with a purified cellulase (endoglucanase) from the fungus *Trichoderma* *viride* (Figure **1A** **and** Video **S1**). Cells were overall resistant to digestion by cellulase, demonstrating that the cell wall of cultured xylem cells is highly recalcitrant. Cells were then pretreated with acidified sodium chlorite (ASC), a classical oxidative chemical treatment known to remove lignin from the cell wall [7,18,26]. In contrast to untreated cells, the secondary wall thickenings of ASC-pretreated cells became thinner during the incubation with cellulase, and by 3 hours they were practically absent (Figure **1B** **and** Video **S1**). Thus, a pretreatment, such as ASC, is necessary to efficiently and rapidly degrade cellulose from the wall of cultured xylem cells using enzymatic means.

### Cell wall degradation in xylem cells can be quantified using polarized light

**Figure 2:**
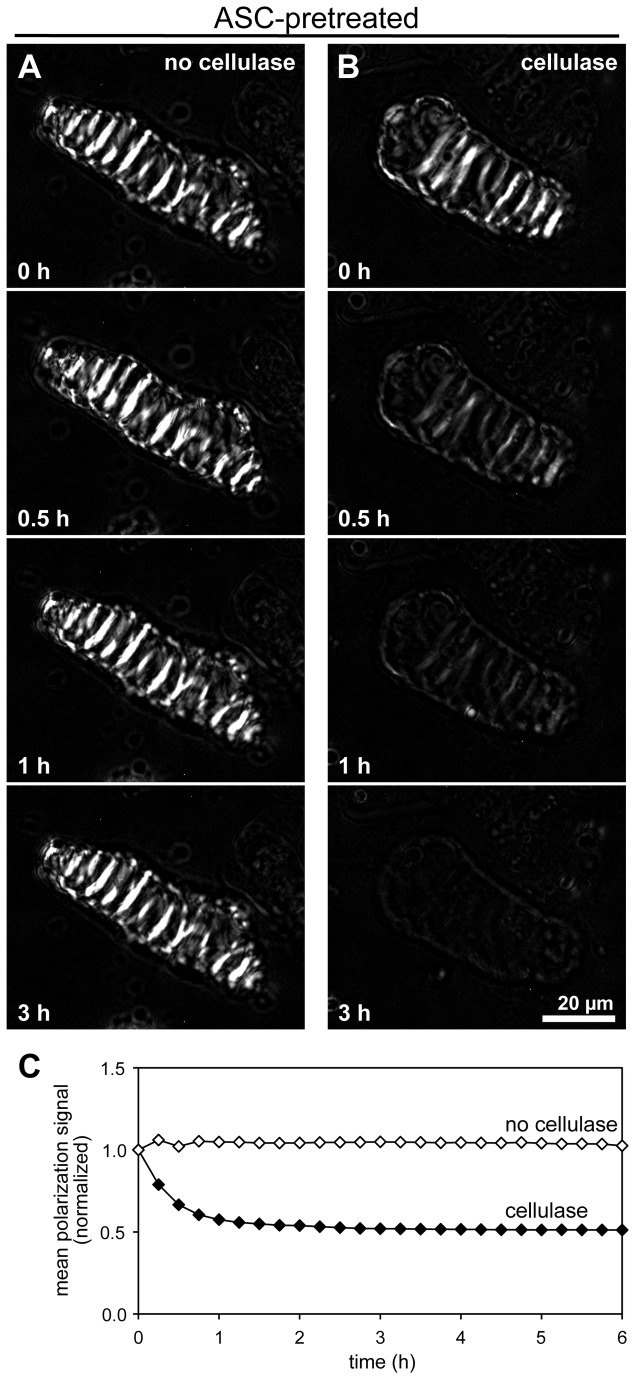
Polarized light indicates the enzymatic digestion of cellulose in secondary walls. Time-lapse polarization microscopy was used to image ASC-pretreated xylem cells incubated in the absence (A) or presence (B) of cellulase. (A) Qualitatively, the polarization signal remains stable during a 3-hour incubation without cellulase. (B) The polarization signal rapidly declines during digestion with cellulase. (C) Quantification of the total polarization signal from the cells in (*A*) and (*B*) reveals a constant signal during incubation with no cellulase (open symbols) and the rapid loss of signal from the cell digested with cellulase (closed symbols). Scale bar=20µm. Also see Video S2.

Since we wanted to quantitatively determine the extent of degradation of the cell wall, we started by using polarized light, which highlights secondary wall thickenings in xylem cells and directly indicates the presence of cellulose [17]. Xylem cells imaged using polarized light microscopy displayed bright bands corresponding to cell wall thickenings (Figure 2). When ASC-pretreated cells were incubated in buffer without cellulase enzyme, we observed that the polarized light intensity signal of cells remained constant (Figure **2A, C** **, and** Video **S2**). However, when similarly pretreated cells were incubated with cellulase, the polarized light signal rapidly decreased, so that it was reduced by approximately 40% within the first hour of incubation and reached nearly background levels by 3 hours (Figure **2B, C** **, and** Video **S2**). These results show that the use of polarized light microscopy can be an effective technique to quantitatively monitor the loss of cellulose, although it is most useful at the level of individual cells.

### *CtCBM3-GFP* fluorescence labeling reveals the rapid digestion of cellulose in populations of pretreated xylem cells

**Figure 3:**
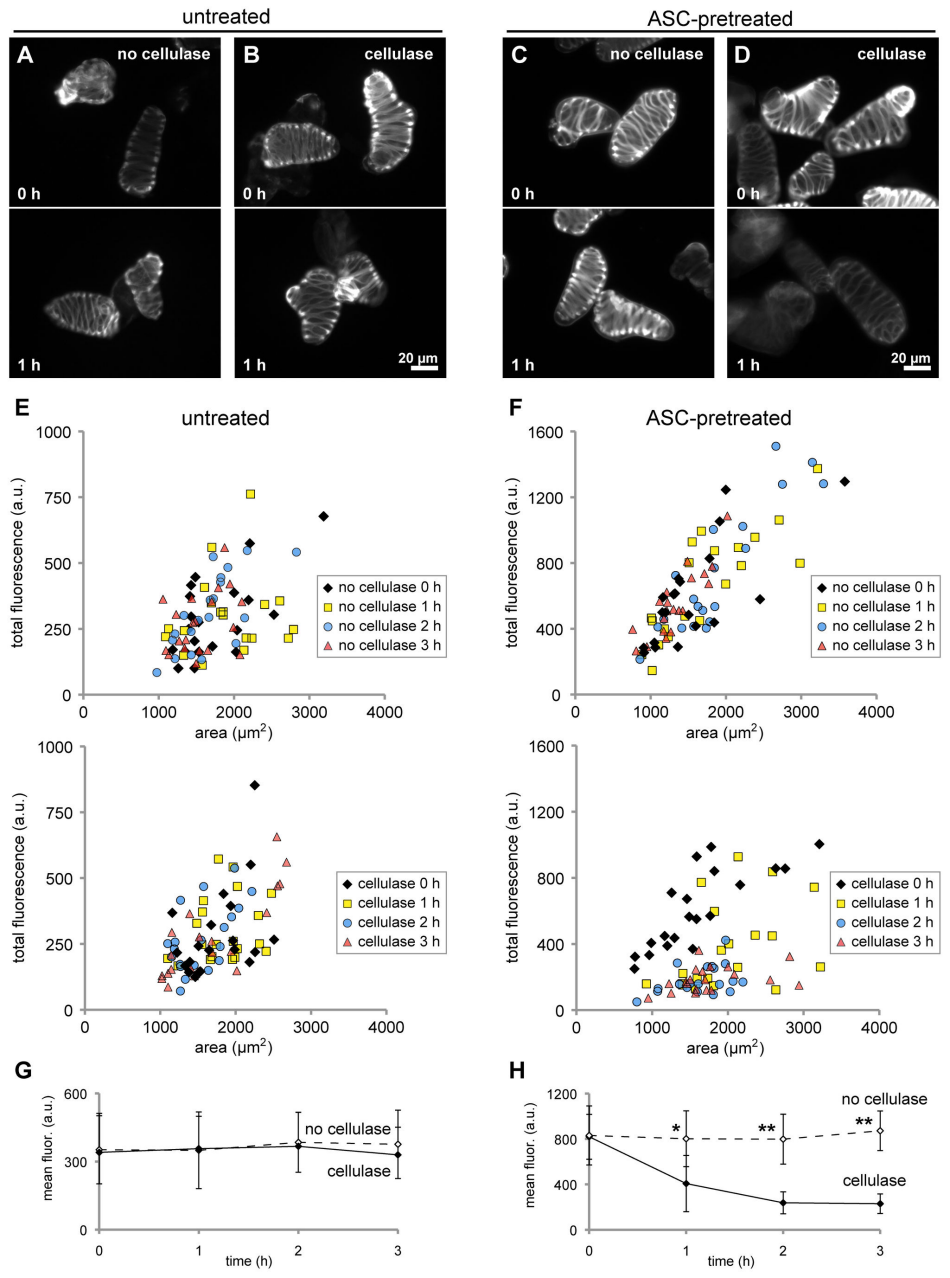
*Ct*CBM3-GFP labeling quantitatively reveals the rapid digestion of cellulose in pretreated cells. (A–D) Fluorescence images show representative xylem cells that were incubated in the absence or presence of cellulase and subsequently labeled with the cellulose-specific *Ct*CBM3-GFP probe. Untreated cells, either undigested (A) or digested with cellulase for 1 hour (B), qualitatively show similar fluorescence levels. Decreased fluorescence levels are evident in cells pretreated with ASC after only 1 hour of digestion with cellulase (D, bottom panel) compared to cells at time 0 (D, top panel) or undigested ASC-pretreated cells (C). (E, F) The total fluorescence values of single xylem cells are plotted as a function of cell area. Four different symbols indicate the time of incubation, as indicated in the inset legends. (E) The fluorescence distributions of untreated cells taken at 1-hour time intervals during a 3-hour incubation without cellulase (top) or with cellulase (bottom) overlap. (F) The fluorescence distributions of ASC-pretreated cells taken at 1-hour intervals during incubation without cellulase (top) are also similar, while the fluorescence levels from cells digested with cellulase (bottom) decreased during the incubation. (G, H) The average of mean fluorescence values of the cells in (*E*) and (*F*) are plotted as a function of time. (G) No significant difference is observed between the mean fluorescence of untreated cells incubated without cellulase (open symbols, dashed line) or with cellulase (closed symbols, solid line). (H) The mean fluorescence of ASC-pretreated cells incubated without cellulase (open symbols, dashed line) or with cellulase (closed symbols, solid line) are significantly different (*p<0.001, **p<0.0001). Error bars=SD. Scale bar=20µm.

Given that the cellulose levels of different cells cannot be directly and accurately compared at a specific time using polarized light, we applied a fluorescence-based labeling strategy to quantify the degradation of cellulose in populations of xylem cells. To specifically label and quantify cellulose content, we used a fluorescently tagged family 3 carbohydrate-binding module (*Ct*CBM3-GFP), which has been shown to bind crystalline cellulose [27]. Based on qualitative observations, untreated cells that were incubated in the absence of cellulase and then labeled with *Ct*CBM3-GFP exhibited similar fluorescence levels after a 3-hour incubation (Figure 3A). To quantitatively compare the result of different treatments, we measured the amount of fluorescence from individual cells and performed statistical comparisons between cell populations. Our measurements first revealed a strong positive correlation between cell fluorescence and area (*r*=0.46 for untreated, undigested cells; Figure **3E** **, top panel**), indicating an increase in cellulose content with larger cell size. This relationship was more pronounced in ASC-treated cells (*r*=0.83 for undigested cells; Figure **3F** **, top panel**), suggesting that cellulose content can be more accurately measured by *Ct*CBM3-GFP after removal of cell wall components using ASC.

Using this quantitative approach, we confirmed that cellulose levels were constant in populations of untreated cells incubated in the absence of cellulase during a 3-hour period (Figure 3E, G). Similarly, the fluorescence levels of untreated cells remained the same when incubated with cellulase, indicating that the enzyme was not able to degrade detectable levels of cellulose (Figure 3**B, E** and **G**). When xylem cells pretreated with ASC were incubated without enzyme, they also maintained similar cellulose amounts as detected by *Ct*CBM3-GFP (Figure 3**C, F** and **H**). In contrast, the fluorescence levels of populations of ASC-pretreated cells gradually decreased when incubated with cellulase. The fluorescent label of the secondary wall thickenings in these digested cells became less pronounced even after 1 hour (Figure 3D), and the fluorescence of each population of cells taken at 1-hour time intervals was significantly different (*p*<0.001) when compared to corresponding cells incubated in buffer without cellulase (Figure 3F, H). Specifically, the mean fluorescence of each population of ASC-pretreated cells was reduced by 50% after 1 hour, 70% after 2 hours, and 74% after 3 hours compared to corresponding undigested cells (Figure 3H). These results show that labeling of populations of xylem cells using a fluorescent cellulose-specific probe can be used to measure the relative amount of cellulose present in the cell wall and also corroborate in a quantitative fashion that pretreatment with ASC significantly improves cellulase digestion.

### Impaired lignification during xylogenesis significantly improves cell wall digestibility by cellulase

**Figure 4:**
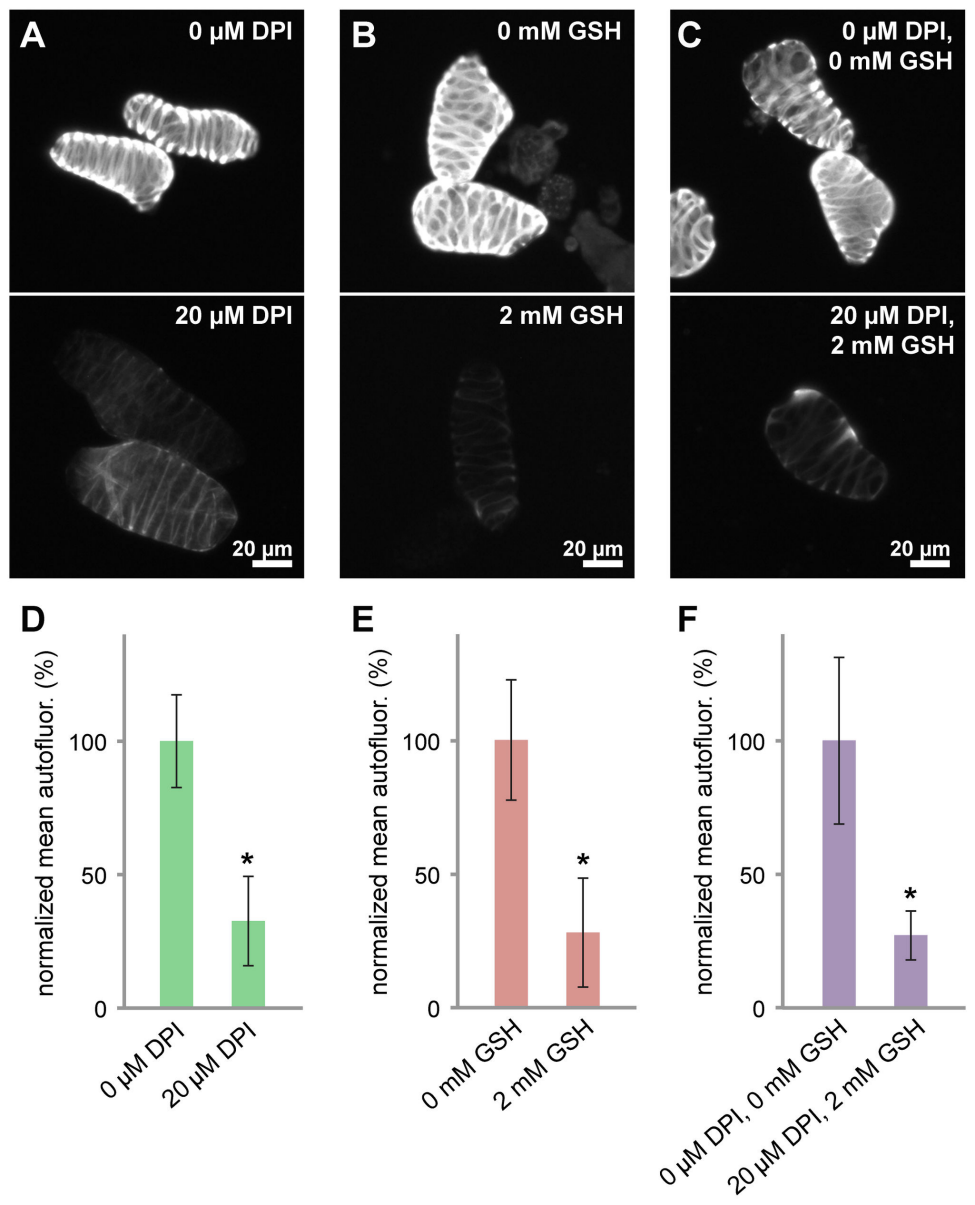
Inhibitors of lignin deposition significantly reduce secondary wall autofluorescence. (A–C) Autofluorescence (512-542 nm) from representative xylem cells is shown, either from control cultures (top panels) or from cultures containing 20 µM DPI, 2 mM GSH, or 20 µM DPI combined with 2 mM GSH (bottom panels). For presentation purposes, images were scaled consistently so that the bottom panels had low, yet noticeable signal levels. Scale bar=20µm. (D–F) Mean autofluorescence values from each population of xylem cells were averaged and normalized to the average from the corresponding control sample. The mean autofluorescence is significantly reduced (*p<0.0001) in treated cells compared to controls. Error bars=SD.

We observed that pretreatment with ASC was necessary to accomplish efficient enzymatic degradation of xylem cells using cellulase. Since ASC is a classical oxidative chemical treatment that primarily removes lignin from the cell wall, we decided to use an alternate approach that would reduce the amount of lignin deposited in xylem cells during their development. Accordingly, we cultured isolated *Zinnia* mesophyll cells in medium containing chemical inhibitors of lignin biosynthesis (diphenyleneiodonium, DPI; and reduced glutathione, GSH) added 3 days after inducing their differentiation into xylem cells in culture. To evaluate the extent of lignification in treated cells, we measured their autofluorescence between 512 and 542 nm, which mainly originates from lignin in the secondary wall thickenings. We found that cell autofluoresce nce was significantly reduced by at least 70% (*p*<0.0001) following treatment with DPI, GSH, or DPI combined with GSH (Figure 4). When we labeled these cells with the lignin dye phloroglucinol, we found fewer stained cells and overall their color was fainter compared to control cells (**data not shown**), which is consistent with the reduction in cell autofluorescence observed.

**Figure 5:**
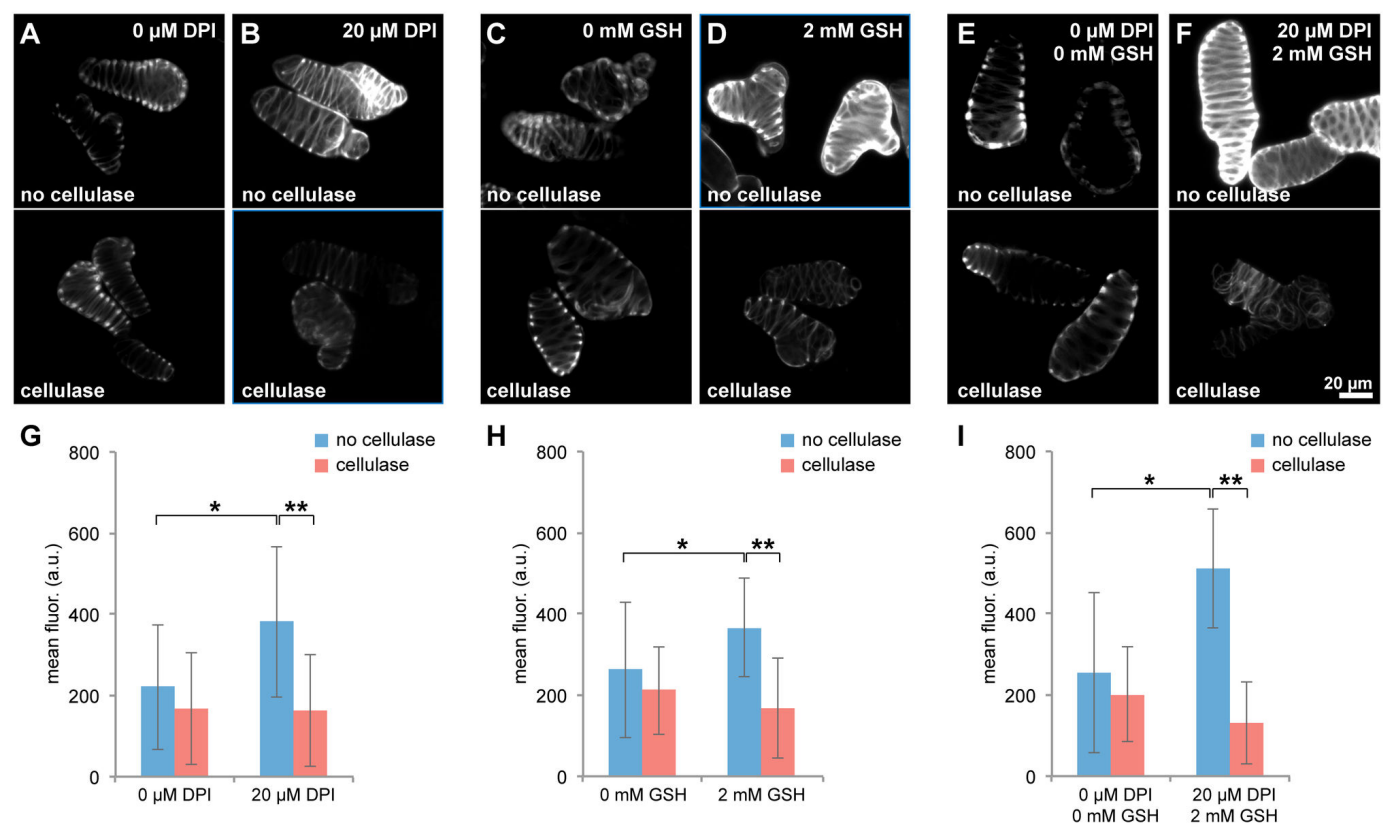
Inhibition of lignin biosynthesis significantly improves the enzymatic removal of cellulose. (A–F) Representative images of xylem cells, either from control cultures (A, C, E), or from cultures containing 20 µM DPI (B), 2 mM GSH (D), or 20 µM DPI combined with 2 mM GSH (F), are shown after incubation without cellulase (top panels) or with cellulase (bottom panels) for 24 hours. All images were corrected for autofluorescence and show fluorescence after labeling cells with *Ct*CBM3-GFP. A reduction in *Ct*CBM3-GFP labeling is evident in cells treated with inhibitors of lignin deposition and then digested with cellulase (B, D, F). Scale bar=20µm. (G–I) The average of mean fluorescence values of populations of xylem cells treated with DPI (G), GSH (H), or DPI combined with GSH (I) and labeled with *Ct*CBM3-GFP are plotted. Mean fluorescence values were corrected for autofluorescence. The fluorescence labeling of undigested xylem cells is significantly increased (*p<0.01) by 73% (G), 40% (H), and 101% (I) in cells treated with inhibitors compared to controls (bracketed blue bars). The mean fluorescence of inhibitor-treated cells is significantly reduced (**p<0.001) by 58% (G), 54% (H), and 74% (I) in digested compared to undigested samples. Error bars=SD.

After confirming that we could generate xylem cell cultures with reduced lignification, we investigated how the accessibility and digestibility of the cell wall were affected. In the experiments described we used *Ct*CBM3-GFP to measure cellulose content; however, as this protein is partly derived from a non-catalytic domain that binds directly to cellulose, it can also be used to examine cell wall accessibility to enzymes of similar size or smaller. Therefore, we compared the binding of *Ct*CBM3-GFP between undigested xylem cells treated with chemical inhibitors of lignin biosynthesis and control samples. We found that inhibitor-treated cells were significantly more fluorescent (*p*<0.01) than the corresponding controls by 73% (DPI), 40% (GSH) and 101% (DPI combined with GSH), showing that treated cells had increased binding of the cellulose probe (Figure **5A–F** **, top panels; and 5G-I, bracketed blue bars**). This result indicates that xylem cells with lower lignin content have increased cellulose accessibility, i.e. more cellulose vulnerable to a potential enzymatic attack.

To determine whether impaired lignification of the cell wall aided the digestibility of cellulose, we compared the difference in *Ct*CBM3-GFP fluorescence between cells incubated with or without cellulase for each inhibitor treatment. In the following experiments, cells were incubated with cellulase for a longer time period (24 hours) in order to clearly detect changes in the cell wall. First, we examined control cells and found that, qualitatively, cells incubated with cellulase maintained similar labeling by *Ct*CBM3-GFP compared to those incubated without any enzyme (Figure 5**A, C** and **E**). We then quantified cell fluorescence and measured a decrease in *Ct*CBM3-GFP labeling in cells digested with cellulase compared to those incubated without cellulase, but the difference was only about 20% and not statistically significant (Figure 5G–I). When we examined cells that had been inhibitor-treated to reduce lignification, a noticeable decrease in *Ct*CBM3-GFP labeling was observed in samples digested with cellulase (Figure 5**B, D** and **F**). Quantitative fluorescence measurements confirmed that inhibitor-treated cells incubated with cellulase had significantly lower amounts of cellulose compared to corresponding cells incubated without cellulase (*p*<0.0001) (Figure 5G–I). Specifically, DPI- and GSH-treated cells exhibited 58% and 54% lower fluorescence labeling, respectively, after cellulase digestion (Figure 5G, H). A more noticeable reduction in *Ct*CBM3-GFP fluorescence was measured in cells treated with DPI and GSH combined, so that fluorescence levels were lower by 74% after cellulase digestion (Figure 5I). These observations suggest that the quantity of lignin present in the cell wall can have a direct negative effect in the digestibility of xylem cells. In summary, our results using ASC and inhibitors of lignin deposition show that enzymatic digestion of cellulose in the cell wall can be dramatically enhanced by lignin depletion.

## Discussion

### Influence of lignin depletion on wall digestibility

The digestibility of lignocellulose from biomass depends on properties attributed to the substrate as well as the hydrolytic enzymes involved. For example, cellulases from diverse microbial species or enzymatic complexes such as cellulosomes have been directly engineered to alter their catalytic activity or stability [28] with the ultimate goal of improving their efficiency in deconstructing biomass. This approach may prove more successful with soluble enzymes, which have recently been shown to have capabilities superior to cellulosomes at accessing and hydrolyzing cell wall cellulose in biomass [29]. In this study, we focused on modifying the substrate, specifically the lignin component of lignocellulose, to examine the effect on the enzymatic hydrolysis of cellulose from the cell wall. Lignin is predominantly thought to hinder the enzymatic hydrolysis of cellulose in the cell wall by two possible mechanisms: by creating a physical barrier that restricts digestive enzyme access [29–31] or by unproductively adsorbing cellulases to prevent their activity [8,9]. Hemicellulose, which is thought to coat and bind to cellulose fibrils [32], may also play a role in decreasing the access of cellulose to hydrolytic enzymes [33,34]. Given the commonly reported inhibitory role of lignin in the enzymatic hydrolysis of lignocellulosic biomass, we depleted lignin in single xylem cells, i.e. tracheary elements, to directly probe the resulting digestibility.

We used two different approaches to decrease lignin content in xylem cells: an oxidative treatment consisting of hot acidified sodium chlorite (ASC) and chemical inhibition of lignin biosynthesis during xylem cell differentiation in culture. ASC is a classical treatment that may be the most prevalent laboratory approach for the removal of lignin from biomass [7,35,36]. This treatment efficiently solubilizes lignin and has been reported to moderately solubilize hemicellulose [35] without a significant decrease in the crystallinity of cellulose [36,37]. Treatment with inhibitors of lignin biosynthesis also results in cell walls with reduced amounts of lignin, but instead of removing lignin at an endpoint, these chemicals impair lignification during the process of secondary wall development in culture [10–12]. Without any delignifying method, we were able to detect some loss of cellulose in cells after a long incubation with cellulase. Nevertheless, the degradation of cellulose was modest, supporting the idea that the cell wall is highly recalcitrant even in single cells. By using the two aforementioned methods to decrease lignin content in the cell wall, we found that cellulase digestion of xylem cells improved significantly. Was this result purely due to reduction in lignin amounts or due to improved access to cellulose? The independent contribution of these two factors has been difficult to address because lignin removal is predicted to affect cellulose accessibility.

### Cellulose accessibility as a function of lignin content

To specifically measure changes in cellulose accessibility, fluorescently labeled probes derived from bacterial cellulases have been successfully used in "pure" cellulosic substrates as well as in corn stover biomass [38–40]. Direct measurements using one of such cellulase-derived probes in corn stover support the notion that increased access of hydrolytic enzymes to cellulose results in more digestible biomass [39]. Interestingly, a recent study that measured cellulose accessibility to cellulase in switchgrass demonstrated that lignin removal alone, without a significant increase in accessibility, does not improve the rate of enzymatic hydrolysis of the substrate [41].

We have previously shown that significant loss of lignin in ASC-pretreated *Zinnia* xylem cells correlates with a striking increase in binding of the cellulose-specific probe *Ct*CBM3-GFP [18]. Furthermore, loss of hemicellulose was detected following ASC, which probably contributed to the enhanced binding of the cellulose probe. Since this probe directly binds to cellulose, it can indicate cell wall accessibility to proteins of similar size or smaller. In the present study, we measured a statistically significant increase in *Ct*CBM3-GFP binding and thus, accessibility to cellulose, in cells whose lignification was blocked during development. Inhibitors of lignin polymerization do not directly alter hemicellulose content, so the increase in *Ct*CBM3-GFP binding found was substantial, yet not as striking as previously observed in ASC-pretreated cells [18].

It has been suggested that even when lignification is selectively targeted in plants, compensatory mechanisms may ensue and affect the characteristics of the cell wall so that there may not be a measurable effect in digestibility [42]. For example, plants with impaired lignin biosynthesis are known to compensate for the lack of lignin by incorporating aldehydes, unusual phenolics, or compounds such as ferulates [43,44], which play a key role in creating cross-linkages within the wall and may restrict the degradation of the cell wall [45]. Instead, we found that the digestibility of inhibitor-treated cells was improved, similar to what was observed in ASC-pretreated cells, and therefore in our system there appears to be no overall compensation resulting from a reduction in lignin biosynthesis.

Given that the inhibition of lignin biosynthesis alters the normal progression of secondary wall assembly, other cell wall matrix components that normally associate with lignin could also become modified or delocalized in inhibitor-treated xylem cells. Hence, we cannot rule out the possible contribution of such factors in reducing the accessibility to cellulose. Nonetheless, our observations collectively showed that cellulose accessibility is a key factor affecting the enzymatic digestibility of the cell wall in *Zinnia* xylem cells. Our results also support the idea that common pretreatments that solubilize polymers coating cellulose fibrils within the cell wall render natural plant substrates more digestible and thus require the use of less enzyme for efficient hydrolysis [8,46].

### Quantification of enzymatic digestion in populations of single cells

The digestion of pretreated xylem cells by cellulase can occur quite rapidly, and the loss of cell wall material is unmistakable using brightfield microscopy. However, this type of microscopic examination is limited to qualitative observations lacking a precise grasp of the dynamics that take place. To quantify the amount of cellulose present in cells we used polarization microscopy, which takes advantage of the birefringent nature of the secondary wall thickenings of xylem cells [17] and allowed us to quantitatively measure the dynamics of cellulose loss in time. Nevertheless, with this technique we cannot directly compare cellulose levels in multiple cells in a single image because different cells in a field of view will have different intensities, depending on their orientation relative to the polarized light source. To take advantage of the power of statistics and quantify cellulose content in multiple cells at the same time, we measured the fluorescence of populations of cells. This method offers the advantage of having discrete objects, in this case individual xylem cells, whose fluorescence can be accurately quantified and directly compared after being labeled with fluorescent probes against specific cell wall polysaccharides, for instance.

Non-catalytic carbohydrate-binding modules (CBMs), which originate from microbial enzymatic complexes, have become attractive probes for the detection of polysaccharides within cell wall structures, particularly because of their intrinsic ability to recognize their targets *in situ* [47]. Currently, a large repertoire of CBMs with specificities toward various polysaccharides that include xylans, mannans, glucomannans, β-(1-3)-linked glucans, mixed-linkage glucans, and cellulose have been identified [27,48–50]. We chose to use the family 3 CBM from the *Clostridium thermocellum cipA* gene, which encodes the scaffoldin subunit of its cellulosome [27]. By fusing the CBM3 domain with GFP, additional labeling steps with CBM-specific antibodies [50,51] were unnecessary. Although a number of monoclonal antibodies have been exploited to detect polysaccharides and proteins within the plant cell wall [52,53], direct cellulose immunolocalization has been difficult, so detection using cellulose-binding peptides or full enzymes from microbes has become a preferred approach [38–41,51,54]. Calcofluor white, a classical fluorescent dye, has been used to detect cellulose in *Zinnia* xylem cells [17], but this dye provides less specificity because it can also bind to non-crystalline cellulose and other structured polysaccharides [51,55]. Taking these factors into consideration, the *Ct*CBM3-GFP labeling strategy used in this study represents a desirable approach, because we could specifically and conveniently detect the accessible cellulose present in the cell wall of individual cells in a single step.

### Final remarks

In summary, after using two different approaches to decrease lignin content in *Zinnia* xylem cells, we observed that these cells became more easily digested by cellulase and concluded that this effect was due to increased access of the enzyme to its target. For decades, *Zinnia* xylem cells or tracheary elements have been used successfully as an *in vitro* cell culture system to understand the process of secondary wall patterning and xylogenesis. Here we show that these cells, which represent a simple lignocellulosic substrate compared to biomass, can be exploited to make quantitative measurements of cell wall deconstruction rather than biogenesis. Finally, fluorescent CBM probes, such as the cellulose-specific *Ct*CBM3-GFP probe used in this study, constitute powerful tools to identify cell wall polymers as well as provide a measure of content and accessibility. Hence, the quantification of accessible polysaccharides in large populations of cultured xylem cells using CBMs is an approach that can be valuable to lignocellulosic biofuel research, particularly if coupled to analytical techniques that may take advantage of the single-cell nature of these *Zinnia* cell cultures.

## Materials and Methods

### Isolation of xylem cells

Cells were cultured and isolated as previously described [18]. Briefly, after growing *Zinnia* seedlings for 2 weeks, the first true leaves were removed and surface sterilized. Mesophyll cells were harvested and cultured as detailed previously [56], except that cells were suspended at a concentration of 10^5^ cells/ml in 6-well plates containing 1 µg/ml of 6-benzylaminopurine and 1 µg/ml of alpha-naphthaleneacetic acid in S-medium. Xylem cells from 7- to 10-day old cultures were separated from undifferentiated and dead cells by density-fractionation using a gradient of plant-tested 72% Percoll (Sigma-Aldrich, St. Louis, MO). The lowest of three distinct bands, which contained the largest number of xylem cells, was collected. Cells were then washed 3 times with double distilled H_2_O by low speed centrifugation using a tabletop Nanofuge (Hoefer Scientific Instruments, Holliston, MA) before additional experimental use.

### Cellulase digestion

Cells were enzymatically digested with a purified endoglucanase from the fungus *T* *. viride* (1,4-(1–1,3,4)-β-D-glucan 4-glucano-hydrolase, Onozuka RS, EC number 3.2.1.4; Sigma-Aldrich, St. Louis, MO), dissolved in 50 mM sodium citrate buffer, pH 5.0. To record cellulase digestion using time-lapse imaging, cells were allowed to attach to 35-mm glass bottom culture dishes (MatTek, Ashland, MA) coated with 0.1% poly-L-lysine (Ted Pella Inc., Redding, CA). After 3 ml of sodium citrate buffer were carefully added to the dish, the temperature (set to 50°C) was allowed to stabilize for approximately 1 hour before addition of enzyme (60 U) or buffer only. For *Ct*CBM3-GFP labeling following digestion, approximately 50 mg of xylem cells (wet pellet) were enzymatically digested using 10 U/ml in sodium citrate buffer by incubation in Eppendorf tubes at 200 rpm, 50°C for 1-24 hours. Aliquots were taken at the specified times and washed 3 times with double distilled H_2_O before labeling with *Ct*CBM3-GFP. Experiments were repeated at least twice, and a single representative experiment was selected for data presentation.

### Image acquisition and analysis

Images were acquired using an inverted DMI6000B Leica Microscope equipped with temperature control components, a CCD camera (DFC360FX), and Leica software (AF6000; JH Technologies Inc., Fremont, CA). Time-lapse images were acquired using a 40X oil-immersion objective, whereas we used a 20X air objective to acquire fluorescence images.

To analyze *Ct*CBM3-GFP labeling or cell autofluorescence in images, we used ImageJ software (http://rsb.info.nih.gov/ij/) to draw close-fitting polygons around single cells and measured their area and mean fluorescence intensity. The total fluorescence was calculated by multiplying the mean intensity by the total number of pixels encompassing each cell. The average of the mean fluorescence of cells was also reported. At least 20 cells were analyzed for each population. In Figure 5, intensity values in images and mean fluorescence values in graphs were corrected by subtracting the mean autofluorescence from corresponding populations of unlabeled cells. Statistical comparisons of fluorescence values were performed using the Mann-Whitney statistical test. The relationships between cell fluorescence and area were reported using Pearson’s correlation coefficient (*r*).

For analysis of polarization signal, we used the same polygon-drawing method and quantified the intensity in each image of the time-lapse sequences. These values were then normalized to the intensity of each cell at time 0.

### Fluorescent labeling of cellulose

*Ct*CBM3-GFP [27] was used as described previously [18,57]. Briefly, cells were incubated with 0.1 µg µl^-1^ of *Ct*CBM3-GFP in 200 µl of 1% BSA, PBS buffer [58] at room temperature for 1.5 hours with gentle mixing, washed 3 times with buffer, and mounted on slides for imaging.

### Treatment with acidified sodium chlorite and inhibitors of lignification

To chemically extract lignin, xylem cells were incubated in acidified sodium chlorite (1% sodium chlorite, 0.14% acetic acid) at 70°C for 20 hours [26] in a single step. Cells were then washed 3 times with double distilled H_2_O before additional manipulation. To inhibit lignification during the differentiation of mesophyll cells into xylem cells, we added diphenyleneiodonium (DPI, dissolved in DMSO; Sigma-Aldrich, St. Louis, MO) and/or reduced glutathione (GSH, dissolved in double distilled H_2_O; Sigma-Aldrich, St. Louis, MO) to cultures in 6-well plates after 3 days in culture [11]. We added the same volume of solvent (DMSO and/or double distilled H_2_O) to control cultures. Culturing was allowed to proceed normally, and xylem cells were enriched by density-fractionation as described above.

## Supporting Information

Video S1 ASC-pretreatment enhances the enzymatic digestion of secondary wall cellulose. Loss of secondary wall thickenings is evident in ASC-pretreated xylem cells (right), while untreated cells appear unaltered (left) during incubation with cellulase. Time-lapse images were collected using brightfield microscopy at 15 min intervals for 3 hours. Also see Figure 1. Scale bar=20µm.
(AVI)

Video S2 Loss of secondary wall cellulose during enzymatic digestion can be monitored by polarization microscopy. The polarized light signal from an ASC-pretreated cell rapidly decreases during incubation with cellulase (right), while the signal from an undigested cell remains stable (left). Time-lapse images were collected at 15 min intervals for 6 hours. Also see Figure 2. Scale bar=20µm.
(AVI)
